# Combining Gram stain and 16S qPCR improved diagnostic accuracy for suspected pneumonia and could become a new metric in the rapid diagnosis of lower respiratory tract infections

**DOI:** 10.1099/jmm.0.001861

**Published:** 2024-08-15

**Authors:** Yunas Panikkaveettil Hamza, Mohamed Ali Ben Hadj Kacem, Naema Hassan Al Molawi, Hadi Mohamad Yassine, Hebah Atef Mohammad AlKhatib, Fatiha Benslimane, Hanan Ibrahim Kh. B. Al-Remaihi, Reham Awni El Kahlout, Basema Ibrahim Ahmed El Kahlout, Hajar Al Khalili, Makiyeh Ahmed Al Khalili, Sanjay H. Doiphode, Emad Bashier Ibrahim Elmagboul, Javed Akhter, Einas A/Aziz Eid Al Kuwari, Peter V. Coyle

**Affiliations:** 1 Hamad Medical Corporation, Doha, Qatar; 2 Annamalai University, Rajah Muthiah Medical College, Chidambaram, India; 3 Biomedical Research Center, Member of QU Health, Qatar University, Doha, Qatar; 4 Wellcome-Wolfson Institute for Experimental Medicine, Queens University, Belfast, UK

**Keywords:** 16S qPCR, 16S sequencing, enhanced Gram stain, Gram stain, MDROs, pneumonia

## Abstract

**Introduction.** The frequency of multidrug-resistant organisms (MDROs) in hospitals and the risk of delaying effective treatment result in the culture of respiratory secretions for nearly all patients with suspected pneumonia. Culture delays contribute to over prescribing and use of broader spectrum antibiotics.

**Gap statement.** The need for improved rapid diagnostics for early assessment of suspected hospital pneumonia.

**Aim.** To validate a new metric, enhanced Gram stain (EGS), to provide a rapid diagnostic test of high diagnostic accuracy that could be assessed in clinical trials of the use of antibiotics in suspected pneumonia.

**Methodology.** Ninety-two residual lower respiratory samples previously tested by culture and Gram stain were re-tested by 16S ribosomal DNA real-time polymerase chain reaction (16S qPCR) and reported as a combined metric with Gram stain termed EGS. The EGS was assessed for diagnostic accuracy, standard performance measurements and correlation against culture. For samples with discordance between culture and EGS, 16S ribosomal DNA whole operon sequencing (16S rDNA WOS) was used for test resolution. An amended EGS (A-EGS was reassessed against culture.

**Results.** Gram stain, 16S qPCR, EGS and A-EGS had respective diagnostic accuracies of 77.01 %, 82.76 %, 84.04 % and 94.19 %. The same platforms had respective correlation with culture of *r* = 0.67, *r* = 0.71, *r* = 0.81 and *r* = 0.89. EGS had the highest negative predictive value (NPV) of 93.18 % (81.99 %–97.62 %). Adding an 16S qPCR result is achievable in most routine laboratories and, combined with Gram stain, could improve early decision-making in patients with suspected hospital pneumonia.

**Conclusion.** EGS could improve early decision-making in patients with suspected hospital pneumonia and could be assessed in clinical trials. The 16S rDNA WOS results in the A-EGS also supported the use of pathogen genomic sequencing in early decision making of suspected pneumonia.

## Introduction

Community-, hospital- and ventilator-acquired pneumonia (CAP, HAP and VAP) definitions use clinical, radiological and location criteria. Because of the frequency of multidrug-resistant organisms (MDROs) in hospitals and the risk of delaying effective treatment, culture of respiratory secretions is recommended for nearly all patients with HAP and VAP [1]. This allows early antibiotic use with early de-escalation if indicated. Gram stain and culture provide early and late diagnostic guidance, respectively; Gram stain also identifies unsuitable samples [2]. Culture delays can lead to over prescribing and over use of broad spectrum antibiotics to avoid poor outcomes [3]. Access to accurate diagnostics is a continuing goal of clinical practice and a research priority [4].

Gram stain is rapid and can be non-inferior to guideline-directed treatment for VAP and able to reduce the use of antipseudomonal and anti-methicillin-resistant *Staphylococcus aureus* agents [5]. Reporting Gram-positive clusters in VAP likewise helped guide anti-*S. aureus* therapy [6], with an earlier meta-analysis study confirming a high negative predictive value (NPV) for Gram stain but a poor kappa agreement with culture [7]. The rapid reporting and relative high specificity of a Gram stain can help initial treatment options. Pan-bacterial 16S qPCR is more sensitive and can help estimate bacterial abundance in a rapid time frame [8], and reporting of cycle threshold (Ct) is familiar since adoption during the pandemic [9].

Microbiome respiratory studies are increasing and use 16S rDNA WOS for describing respiratory population dynamics. Pneumonia is a dysbiosis with culture-positive samples reporting high bacterial DNA burdens (taxon abundance) and low bacterial community diversity [10]. High cost, lack of commercial automated platforms, lack of treatment thresholds and poor bioinformatics support in clinical practice are obstacles to adoption. Current accreditation and regulatory requirements also pose significant challenges to bringing 16S rDNA WOSbased assays to market.

A research priority is to see whether non-invasive or invasive sampling with culture can reduce antibiotic use, antibiotic resistance and direct and indirect costs and improve clinical outcomes without triggering adverse events [11]. Culture is slow, while the quicker results from Gram and 16S qPCR might provide a better approach, and both are proven on all types of respiratory samples. These include (a) *non-invasive*: sputum (spontaneous and induced), nasotracheal suction and endotracheal aspiration (ETA); (b) *invasive*: protected specimen brush (PSB), bronchoalveolar lavage (BAL), blind BAL, bronchial wash (BW) and lung biopsy. Treatment thresholds include PSB > 10^3^ colony forming units (c.f.u.)/ml, BAL > 10^4^ c.f.u. ml^−1^ and ETA > 10^5^ c.f.u. ml^−1^.

The research aim of this study was to test if combined reporting of 16S qPCR and Gram stain could improve early confirmation or exclusion of infection with potential for better antibiotic use. A new metric, enhanced gram stain (EGS), was developed to allow joint reporting of both assays. Performance of the separate and combined assays against culture was assessed. For discordant samples, 16S rDNA WOS was performed and allowed an amended EGS (A-EGS) score to be separately assessed. Correlation with culture was undertaken for the four metrics used in the study.

## Methods

### Study samples

Residual routine samples between November 2018 and March 2019 previously tested for respiratory viruses and stored at −80 °C were available for inclusion. Samples were selected where a paired sample had been co-tested on the same day by Gram stain and culture. Age and gender of the cohort were established from computer records.

### DNA extraction

Extraction used the EZ Virus Mini Kit v. 2.0 (QIAGEN GmbH, Hilden, Germany) following the manufacturer’s instructions, with sample input of 600 µl and the elution volume of 60 µl. The extracted genomic DNA (gDNA) was stored at −80 °C for subsequent processing.

### 16S qPCR

Primer-probe combinations (Applied TaqMan Gene Expression Assays, Life Technologies Corp, Paisley, UK) were based on a previously reported pan-bacterial 16S qPCR assay [12] with the probe aligned to positions 1002 to 1024 of the 16S rDNA gene (Table 1). An RNAse-P Internal Control was included. TaqPath 1-Step Multiplex Master Mix reaction pools were cycled on a QuantStudio 5 (Applied Biosystem) using Uracyl N-Glycosylase treatment by incubation at 25 °C for 2 min, reverse transcription incubation at 50 °C for 15 min and enzyme activation at 95 °C for 2 min, 40 cycles at 95 °C for 3 s and 60 °C for 30 s each. Final primer and probe concentrations were 0.4 and 0.1 µM, respectively, in a final reaction volume of 20 μl.

**Table 1.** 16S Real-time Polymerase Chain Reaction (16S RT-qPCR) primer and probe sequences

Target	Primer/probes	Sequence (5′–3′)
16S	16S-F-1A	TGGAGCATGTGGTTTAATTCGA
16S	16S-R-1	TGCGGGACTTAACCCAACA
16S	16S 1P	FAM-CACGAGCTGACGACARCCATGCA-BHQ
RNAse-P	RnP-1A	AGATTTGGACCTGCGAGCG
RNAse-P	RnP-1B	GAGCGGCTGTCTCCACAAGTA
RNAse-P	RnP-1P	VIC-TTCTGACCTGAAGGCTCTGCGCG-BHQ

Primers and probes used for 16S qPCR on lower respiratory tract samples.

### Whole operon sequencing of the 16S rDNA locus

Extracted gDNA was quality checked using an Agilent Fragment Analyser, yield quantified with High Sensitivity DNA kit and Qubit 2.0 Fluorometer (Thermo Fisher Scientific) and made up to 10 ng by buffer modification. It was sequenced using the 16S Barcoding Kit SQK-16S024 (Oxford Nanopore Technologies, Oxford, UK). The full 16S hypervariable region (V1–V9) was amplified through 35 cycles of amplification in a 50 μl final volume with NEB Next LongAmpHot start 2x Master Mix (New England Biolabs, Ipswich, MA, USA) as the PCR polymerase reagent mixture. The concentration of each purified 16S V1–V9 amplicon was confirmed with an Invitrogen Qubit 4 fluorometer (Thermo Fisher Scientific, Waltham, MA, USA), and the samples were pooled with 50–100 ng of DNA and incubated with 1 µl of Rapid Adapter at room temperature for 5 min. The prepared DNA library (11 µl) was mixed with 34 µl of sequencing buffer, 25.5 µl of loading beads and 4.5 µl of water and loaded onto the R9 flow cell (FLO-MIN106D; ONP, Oxford, UK.)

The protocol was initiated using the MinKNOW ONT software (23.07.12 bionic) and performed using a MinION Flow Cell (R9.4.1, FLO-MIN-106D, ONP, Oxford, UK) on a MinION sequencer (MinION Mk1C). Fast base calling used the MinKNOW ONT software (23.07.12 bionic) in real-time. FASTQ reads were collected 5–8 h after the start of sequencing. De-multiplexing and adapter trimming were performed automatically in real-time, and each FASTQ file obtained per ONT-barcode data was used for downstream analysis (wf-metagenomics workflow). Two different sub-workflows, kraken2 and minimap2, were accessed through EPI2ME lab (EPI2ME V5.1.8 Oxford Nanopore Technologies).

### Reporting of Gram stain and culture results

Gram stain and culture results for lower respiratory tract samples were reported ranked as negative, scanty, moderate or profuse as is common practice. For the study, the ranks were arbitrarily scored 0–3, respectively. Quantitative results of <10^4^ colonies/ml were ranked as scanty (score 1) and those ≥10^4^ colonies/ml as moderate (score 2). Gram stains also reported specimens unsuitable for culture where the laboratory recorded epithelial cells, a marker of upper airway contamination. Culture reported a small number of samples as normal flora along with the negative result.

### Setting a 16S qPCR cut-off threshold

The strength of agreement between culture and 16S qPCR at different CTs was assessed using kappa scores; scanty, unsuitable and normal flora were excluded to reduce stochastic noise. The threshold where agreement was lost was used to define the positive-negative cut-off for assessing the 16S qPCR performance against culture. Ct values of positive and negative results were also ranked and arbitrarily scored 0 to 3 to allow correlation with culture (Table 2).

**Table 2.** Scoring scheme for Gram stain and 16S qPCR

16S qPCR* Ct† value	16S score	Gram stain result	Gram score
≤21	3	Profuse	3
22–24	2	Moderate	2
25	1	Scanty	1
≥26	0	Negative	0

Ranking and assigned score for 16S qPCR and Gram stain results.

*qPCR = Real-time polymerase chain reaction.

†Ct = Cycle threshold value.

### Performance of Gram stain and 16S qPCR against culture

The sensitivity, specificity, Positive Predictive Value (PPV), NPV and diagnostic accuracy of Gram stain and 16S qPCR were determined against culture. For the analysis, all culture results, including those reported as scanty and normal flora, were included to match real-world conditions.

### Performance of the EGS against culture

Results from Gram stain and 16S qPCR were combined using the respective scores of 0 to 3 (Table 2). For each specimen, individual Gram stains were reported as Gram-positive cocci (GPC), Gram-positive bacilli (GPB), Gram-negative cocci (GNC) and Gram-negative bacilli (GNB) using the scanty to profuse nomenclature and scored as in Table 2. The highest individual score was used as the Gram score for calculating the EGS. The EGS combined the sum of the respective Gram and 16S qPCR scores and reported over a scale of 0 to 6, with scores of ≥1 reported as positive to determine sensitivity, specificity, PPV, NPV and diagnostic accuracy against culture. All culture results, including those reported as scanty and normal flora, were included to best match real-world conditions.

### Amended EGS: resolution of discordant results

Discordant results between EGS and culture were resolved by 16S rDNA WOS. Using a previously validated protocol based on comparing culture positive, culture negative, culture unsuitable and samples with normal flora against bacterial abundance by 16S rDNA WOS, a discordant result was defined as positive if a respiratory pathogen was confirmed in the top three species by abundance and negative if only commensal bacteria were identified. The A-EGS score was then used for recalculation of performance characteristics against culture.

### Correlation against culture

Correlation coefficients with 95 % confidence intervals were calculated against culture for each of the four assay platforms.

### Statistical analysis

Descriptive statistics was presented as median and inter-quartile (IQR) range for continuous variables and number and percentages for categorical variables. Statistical analysis was undertaken using free online statistics calculators. MedCalc for Windows, version 22.007 (MedCalc Software, Ostend Belgium), was used for (a) kappa inter-rater agreement scores (https://www.medcalc.org/calc/kappa.php) and (b) sensitivity, specificity, positive predictive value, negative predictive Value and diagnostic accuracy (https://www.medcalc.org/calc/diagnostic_test.php) of each assay against culture; 95 % confidence intervals were reported. Median and IQR for age and Ct values were calculated using BoxPlotR available at *http://shiny.chemgrid.org/boxplotr/*. Pearson’s correlation coefficient reporting 95 % confidence intervals of the respective four assay platforms against culture was performed using Statistics Kingdom, available at Correlation Confidence Interval Calculator (statskingdom.com).

## Results

### Study population

The study cohort included 54 male and 21 female patients aged from 21 years to 93 years with a median age of 50 years (IQRs: 36.5 years–68 years). The samples included 45 BALs, 38 BWs, 6 Sputa and 3 ETAs. Five samples were found to be unsuitable for culture by Gram stain. All test results for Gram stain, culture, 16S qPCR, EGS, and A-EGS are presented in Table S1, available in the online Supplementary Material (S!).

### Cut-off threshold for 16S qPCR against culture

A set of 66 samples ranked as negative, moderate or profuse growth were tested with Ct values reported over a range from Ct 15 to 27 with a median value of Ct 25 (IQR: 22–26). The thresholds that gave the best inter-rater agreements were Ct ≤ 24 and Ct ≤ 25 (Table 3), with a loss of agreement at Ct26. Samples with Ct ≥ 26 were classified as negative for performance analysis.

**Table 3.** Inter-rater kappa scores for establishing a cut-off threshold for 16S qPCR

16S qPCR* Ct† value	Kappa score (95 % CI‡)
≤23	0.74 (0.57−0.0.91)
≤24	0.81 (0.66−0.0.95)
≤25	0.78 (CI: 0.63−0.0.93)
≤26	0.14 (0.03−0.0.25)

Inter-rater agreement kappa scores against culture for Ct thresholds from Ct 23 to Ct 26.

*16S qPCR = 16S real-time polymerase chain reaction.

†Ct = Cycle threshold value.

‡CI = Confidence interval.

### Performance of Gram stain, 16S qPCR, EGS and A-EGS against culture

Performance characteristics are shown in Table 4. Gram stain was the least sensitive, 68.29 % (51.91 %–81.92 %), but with better specificity at 84.78 % (71.13 %–93.66 %) than either the 16S qPCR or EGS. The increased sensitivity of 16S qPCR gave EGS the highest NPV at 93.18 % (81.99 %–97.62 %) but with a lower specificity than Gram stain at 77.36 % (63.79 %–87.72 %). The A-ECG achieved >90 % performance in all categories.

**Table 4.** Assay performance against culture

Assay	Sensitivity (95% CI)	Specificity (95% CI)	Positive predictive value (95% CI)	Negative predictive value (95% CI)
Gram stain	68.29 % (51.91 %–81.92 %)	84.78 % (71.13 %–93.66 %)	80.01 % (66.22 %–89.09 %)	75.01 % (65.32 %–82.70 %)
16S qPCR*	90.24 % (76.87 %–97.28 %)	76.09 % (61.23 %–87.41 %)	77.08 % (66.55 %–85.05 %)	89.74 % (77.28 %–95.75 %)
EGS†	92.68 % (80.08 %–98.46 %)	77.36 % (63.79 %–87.72 %)	76.01 % (65.65 %–83.99 %)	93.18 % (81.99 %–97.62 %)
A-EGS‡	97.5 % (86.84 %–99.94 %)	91.3 % (79.21 %–97.58 %)	90.7 % (79.24 %–96.14 %)	97.67 % (85.82 %–99.66 %)

Performance of Gram stain, 16S qPCR, EGS and A-EGS against culture.

*16S qPCR = 16S real-time polymerase chain reaction.

†EGS = Enhanced Gram stain.

‡A-EGS = Amended-enhanced Gram stain.

### Diagnostic accuracy, inter-rater kappa agreement and correlation against culture

Increasing diagnostic accuracy was noted against culture for each category (Table 5). Gram stain had the lowest diagnostic accuracy at 77.01 % (66.75 %–85.36 %) and poorest correlation with culture while A-EGS had the highest respective values, followed by EGS.

**Table 5.** Diagnostic accuracy, inter-rater kappa agreement and correlation of Gram stain, 16S qPCR, EGS and A-EGS against culture

Assay	Diagnostic accuracy (95% CI)	Inter rater kappa score (95% CI)	Correlation coefficient (95% CI)
Gram stain	77.01 % (66.75 %–85.36 %)	0.53 (0.36 to 0.71)	*r* = 0.67 (0.54, 0.77)
16S qPCR*	82.76 % (73.16 %–90.02 %)	0.66 (0.5 to 0.81)	*r* = 0.71 (0.59, 0.81)
EGS†	84.04 % (75.05 % to 90.78 %)	0.65 (0.5 to 0.81)	*r* = 0.81 (0.71, 0.86)
A-EGS‡	94.19 % (86.95 %–98.09 %)	0.88 (0.78 to 0.98)	*r* = 0.89 (0.83, 0.93)

Respective diagnostic accuracy, inter rater kappa agreement and correlation of Gram stain, 16S qPCR, EGS and A-EGS against culture.

*16S qPCR=16S real-time polymerase chain reaction.

†EGS=Enhanced Gram stain.

‡A-EGS=Amended-enhanced Gram stain.

### Discordant and unsuitable samples

There were 15 discordant results between the EGS and culture (Table S1). EGS classified 12 culture-negative samples as positive, and these resolved as commensal bacteria [8], *Haemophilus influenzae* [2], *Proteus mirabilis* [1] and *Streptococcus pneumoniae* [1] by 16S rDNA WOS, respectively. Of the three discordant samples reported positive by culture and negative by EGS, two were available for sequencing. Neither agreed with the culture results. One sample reported as scanty *Elizabethkingia meningoseptica* was reported as commensal bacteria, and the second had a scanty growth of *H. influenzae* that reported a *S. pneumoniae* by sequencing 16S rDNA WOS. The five samples reported as unsuitable for culture based on epithelial cell contamination were all confirmed as commensal flora.

## Discussion

This proof-of-concept study aimed to see if a new metric, EGS, could provide an early and cost-effective indicator of suspected hospital pneumonia; it also allowed validation of the performance of the four assay platforms against culture. A secondary aim was to determine if 16S rDNA WOS could improve PPV and NPV. The findings supported an improvement in results that were reportable within 2 h of receipt, at marginal increased costs. They also suggest that a bigger role for genomic sequencing in routine practice should be explored.

Conway Morris *et al*. [8] demonstrated the potential for pan-bacterial 16S qPCR for rapid confirmation of VAP, reportable in <6 h. The current study found a similar but less sensitive performance of 16S qPCR, but when combined with Gram stain, it improved the diagnostic accuracy and, importantly, the NPV, the latter to 93.18 %(81.99 %–97.62 %). This could support safe delay in starting antibiotic treatment in most patients where judged suitable on clinical grounds. The A-EGS improved both NPV (97.67 % (85.82 %–99.66 %)) and PPV (90.7 % (79.24 %–96.14 %)) suggesting that 16S rDNA WOS could have a role in the early decision to use antibiotics treatment.

A predictable weakness of 16S qPCR was the generation of false-positive results due to contamination by airway flora. Sequencing confirmed commensal bacteria in 8 of the 12 samples positive by 16S qPCR and respiratory pathogens in four others. The scores and culture ranking of the latter four samples confirmed weak positive results with a risk of stochastic noise, and this should be factored into any clinical decisions taken in advance of culture. The results also supported the use of EGS for earlier decision-making linked to its high NPV. The A-ECG improved all diagnostic markers and could further improve earlier decision-making before culture. Currently, it is feasible to report sequence results in <8 h, but technical complexity would make that difficult for many labs and emphasizes the need for better designed platforms for this type of turn-around time. In this study, Gram stain detected only about 50 % of unsuitable samples and the need for improved ways to assess sample quality.

While the use of routinely reported real-world results was a strength of the work, there were weaknesses as would be the case with any observational, single-centre study, but the clinical units and laboratories involved were, respectively, Joint Commission International and College of American Pathologists accredited which supports good clinical and laboratory practice. The extraction protocol also did not include a mechanical disruption step which could have improved assay performance [13]. Also, resolving discordant results through a process of sequencing to select whether one of the top abundant bacterial species was commensal or a respiratory pathogen was based on preliminary validation of this approach which could be strengthened. For example *Proteus mirabilis* was classified as a respiratory pathogen for this analysis which was discordant with the EGS result. Also, there was no attempt to place the laboratory findings in their clinical context, which would in practice be critical to the effective use of the new metric.

### Conclusion

EGS is a new metric that provides a rapid, simple and cost-effective result with a high NPV that could support better early use of antibiotics in patients with suspected hospital pneumonia; intervention trials should be supported to demonstrate this in clinical practice. The increased performance of the A-EGS improved both PPV and NPV and could further improve early management decisions, suggesting a bigger role for pathogen genomic sequencing in the early diagnosis of suspected pneumonia. Commercial platforms should be designed to make this possible and affordable.

## Supplementary material

10.1099/jmm.0.001861 Uncited Table S1.

## Abbreviations

A-EGS: amended EGS
BAL: bronchoalveolar lavage
BW: bronchial wash
CAP: Community-acquired pneumonia
c.f.u.: colony forming unit
EGS: Enhanced Gram stain
ETA: endotracheal aspiration
gDNA: genomic DNA
GNB: Gram-negative bacilli
GNC: Gram-negative cocci
GPB: Gram-positive bacilli
GPC: Gram-positive cocci
HAP: Hospital-acquired pneumonia
IQR: Interquartile range
MDROs: multidrug-resistant organisms
NPV: negative predictive value
PPV: Positive Predictive Value
PSB: Protected specimen brush
16S qPCR: 16S ribosomal DNA real-time polymerase chain reaction
16S rDNA WOS: 16S ribosomal DNA whole operon sequencing
VAP: ventilator-acquired pneumonia
